# Comparison of transradial and transfemoral access for transcatheter arterial embolization of iatrogenic renal hemorrhage

**DOI:** 10.1371/journal.pone.0256130

**Published:** 2021-08-20

**Authors:** Chuanwu Cao, So-Yeon Kim, Gun Ha Kim, Ji Hoon Shin, In Chul Nam, Meshari Alali, Hee Ho Chu, Heung-Kyu Ko

**Affiliations:** 1 Department of Radiology, The Tenth People’s Hospital, Shanghai, China; 2 Department of Radiology and Research Institute of Radiology, University of Ulsan College of Medicine, Asan Medical Center, Seoul, Korea; 3 Department of Radiology, Gyeongsang National University College of Medicine and Gyeongsang National University Hospital, Changwon, Korea; 4 Department of Radiology, Majmaah University, Almajmaah, Saudi Arabia; Ohio State University Wexner Medical Center Department of Surgery, UNITED STATES

## Abstract

**Background:**

There are few reports of renal artery embolization (RAE) via transradial access (TRA) for renal hemorrhage, and none have compared outcomes of RAE via TRA and transfemoral access (TFA). The objective was to compare technical and clinical outcomes in patients undergoing RAE via TRA or TFA for iatrogenic renal hemorrhage.

**Materials and methods:**

This study included 45 RAE procedures (16 TRA and 29 TFA) for iatrogenic renal hemorrhage in 43 patients performed at a tertiary referral center between October 2018 and December 2020. Information regarding underlying diseases, coagulation status, angiographic and embolization procedure details, technical and clinical successes, and complications were retrospectively evaluated.

**Results:**

There were no differences in demographics, underlying diseases, updated Charlson comorbidity scores, angiographic findings, and volume of contrast material between the TRA and TFA groups. By contrast, prothrombin time and international normalized ratio were significantly lower in the TRA than in the TFA group. Embolic materials differed significantly in the two groups. Procedure duration, fluoroscopy time, digital subtraction angiography number, and dose area product were slightly lower in the TRA than in the TFA group, but the differences were not statistically significant. Technical and clinical success rates in the TRA and TFA groups were 100% and 96.6%, and 100% and 96.6%, respectively. No patient in either group experienced procedure-related complications during a 4 week follow-up period.

**Conclusion:**

RAE via TRA in the management of iatrogenic renal hemorrhage was safe and feasible, with similar procedure duration and radiation exposure to RAE via TFA. TRA may be an acceptable alternative to TFA in these patients.

## Introduction

Renal vascular injuries are the main complications of partial nephrectomy and percutaneous procedures, and require immediate diagnosis and treatment. Transcatheter arterial embolization for renal hemorrhage, including iatrogenic renal hemorrhage, is now considered the treatment of choice, as it is both feasible and minimally invasive, while providing effective hemostasis [1, 2]. Renal artery embolization (RAE) via transfemoral access (TFA) has become a standard method, as it is both safe and effective [1–3].

The number of transradial access (TRA) procedures has increased in patients undergoing visceral interventions, as TRA was found to enhance safety in patients with coagulopathy, as well as to result in early ambulation and discharge [4–7]. TRA was shown to be a feasible and safe alternative to TFA for renal interventions [8, 9]. Stenting and percutaneous transluminal angioplasty have been the most frequent renal intervention procedures performed via TRA [8, 9]. Few studies, however, have assessed RAE via TRA for renal hemorrhage [10], and none, to our knowledge, have compared outcomes of RAE via TFA and TRA. The present study therefore compared technical and clinical outcomes in patients undergoing RAE via TFA or TRA for iatrogenic renal hemorrhage.

## Materials and methods

The study was approved by the institutional review board of Asan Medical Center, which waived the requirement for informed consent due to the retrospective nature of this study.

### Patients

The cohort for this study consisted of 43 consecutive patients who underwent RAE for iatrogenic renal hemorrhage via TRA or TFA at Asan Medical Center, a tertiary medical center in Seoul, Korea, between October 2018 and December 2020. Because of the risk of hand ischemia, Barbeau type D waveform was regarded as a contraindication for TRA in patients with radial obstructive complications secondary to poor ulnar compensation [5]. The minimal radial artery diameter for study inclusion was 1.6 mm. Patients’ electronic medical records and information from the picture archiving and communication system were reviewed retrospectively to obtain information on their demographic characteristics, underlying diseases, and coagulation status (Table 1). Patients’ comorbidity scores were calculated using the updated Charlson comorbidity index [11]. Other factors recorded included angiography findings, embolization details, procedure duration, fluoroscopy time, digital subtraction angiography numbers, dose area product (DAP), technical and clinical success rates, creatinine concentrations before and after RAE, and complications.

**Table 1:** Patient demographic characteristics, underlying diseases, comorbidity scores, and coagulation status.

	TRA (n = 16)	TFA (n = 27)	*P* value
Age, year	56.06±18.42	57.33±12.61	0.790
Male:Female	14:2	19:8	0.276
Height, cm	1.69±0.06	1.66±0.1	0.249
Weight, kg	69.62±9.54	68.63±13.34	0.797
BMI, kg/m^2^	24.24±2.29	24.77±3.61	0.603
Systolic blood pressure, mmHg	132.13±24.13	128.74±14.55	0.616
Diabetes mellitus	8 (50)	5 (18.52)	0.043
Coronary artery disease	1 (6.25)	0 (0)	0.372
Smoking	5 (31.25)	7 (25.93)	0.737
Chronic lung disease	1 (6.25)	0 (0)	0.372
Chronic kidney disease	1 (6.25)	0 (0)	0.372
eGFR	69.38±26.55	75.98±26.95	0.263
Updated Charlson comorbidity score	0.69±1.74	1.04±1.68	0.356
PT (sec)	12.36±0.63	13.94±3.39	0.037
INR	1.03±0.05	1.21±0.27	0.004

Results are reported as mean ± standard deviation or as number (%).

**Abbreviations**: BMI, body mass index; eGFR, estimated glomerular filtration rate; INR, international normalized ratio; PT, prothrombin time; TFA, transfemoral access; TRA, transradial access.

### RAE technique

For RAE via TRA, the radial artery was evaluated before the procedure by measuring its diameter and by performing the Barbeau test. The left arm was positioned at 75–90°, almost perpendicular to the table. The left radial artery was accessed with a 5-Fr hydrophilic sheath (Prelude Ease, Merit Medical, South Jordan, UT, USA), using the Seldinger technique. A bolus of 200 μg nitroglycerine and 2.5 mg verapamil was administered through the sheath over 20 seconds to prevent spasm of the radial artery. The relevant renal artery was accessed via a 5-F angled catheter (Performa Transradial Angiographic Catheter, 125 cm, Merit Medical) and a 0.035 inch hydrophilic guidewire (Terumo, Tokyo, Japan). The relevant renal artery was evaluated by digital subtraction arteriography to determine the bleeding focus. The culprit artery was cannulated and embolized using a 2.0-Fr 150 cm microcatheter (Merit Pursue microcatheter, Merit Medical) and a 0.014 inch microwire (True Form, Merit Medical). After the procedure, a TR band (Terumo) was used to achieve patent hemostasis. The balloon was deflated 30 minutes after removal of the sheath.

RAE via TFA was performed using a procedure similar to those previously reported [1, 12]. The common femoral artery was punctured, and a 5-Fr vascular introducing sheath was inserted. A 5-Fr angiographic catheter (Cobra Catheter, Roche Hepatic catheter; Cook Medical, Bloomington, IN, USA) was introduced over a 0.035 inch hydrophilic guidewire (Radifocus, Terumo), and the relevant renal artery was evaluated by digital subtraction arteriography to determine the bleeding focus. The culprit artery was cannulated and embolized using a 2–2.2-Fr microcatheter (Progreat, Terumo). After the procedure, the puncture site was manually compressed by applying Clo-Sur PLUS PAD (Medtronic Vascular, Santa Rosa, CA, USA) for 5 minutes.

All procedures were performed in a dedicated angiography suite with a flat panel detector (Siemens, Artis, Erlangen, Germany). The use of fluoroscopy was minimized, with the pulse rate varying between 4 and 7.5 pulses per sec, depending on the operator’s preference. Angulation projections were not routinely used.

Angiographic findings of active bleeding could indicate contrast extravasation, a pseudoaneurysm, an arteriovenous shunt, or an arterial cut-off. After the bleeding focus was identified, RAE was performed using microcoils (Hilal, Tornado or Nester; Cook Medical), gelatin sponge particles (Spongostan; Johnson & Johnson, Gauteng, South Africa), PVA particles (Contour; Boston Scientific), NBCA (Histoacryl; Braun, Sempach, Switzerland), vascular plugs (Amplatzer Vascular Plug; AGA Medical, Golden Valley, MN, USA), or their combinations.

### Follow-up and definitions

Patients were followed up 4 weeks after the procedure by Doppler ultrasound examination of the access site. All complications, including access site complications (hematoma, pseudoaneurysm, and vessel occlusion), were recorded. Minor complications were defined as those not requiring additional treatment or hospitalization overnight for observation. Major complications were defined as the requirement for therapy with minor hospitalization (<48 h), major therapy, prolonged hospitalization for >48 h, or an unplanned increase in the level of care, as well as permanent adverse sequelae or death [13].

Procedure duration was defined as the interval from patient placement in the supine position on the examination table until the end of the procedure. Technical success was defined as complete exclusion of the bleeding focus, which was no longer opacified on immediate post-embolization angiography. Clinical success was defined as resolution of presenting symptoms without the need for further intervention or surgical procedure.

### Statistical analysis

Continuous variables were reported as mean ± SD and compared by Student’s t-tests or Wilcoxon rank-sum tests, where applicable. Categorical variables were reported as percentages and compared by chi square tests or Fisher’s exact tests, as warranted. P-values < .05 were considered statistically significant.

## Results

**Fig 1:**
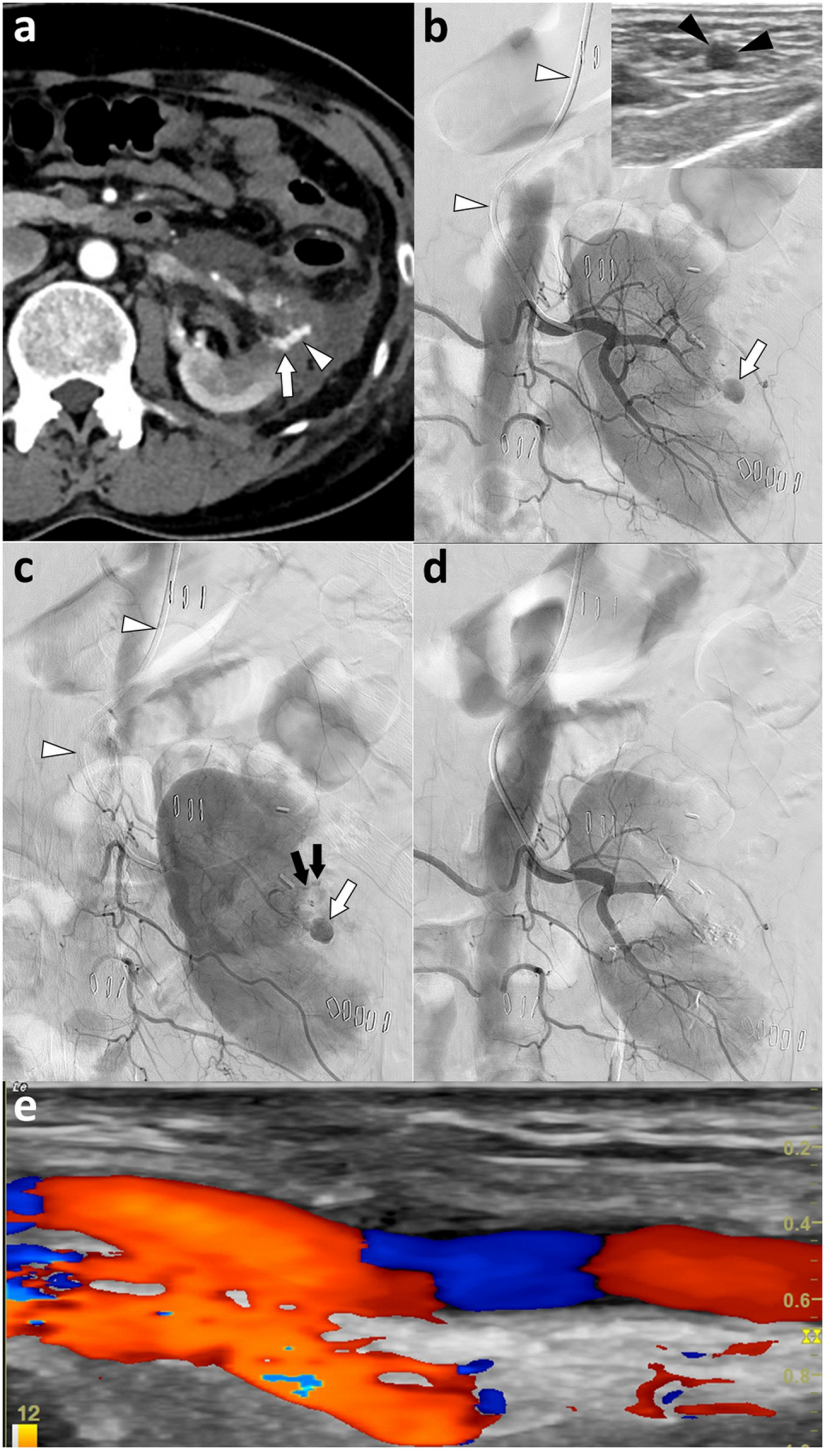
Transradial access for embolization of renal hemorrhage after partial nephrectomy performed 5 days earlier for renal cell carcinoma in a 49-year-old woman. Left radial artery diameter was 2.2 mm, and Barbeau test was type A. (a) Contrast-enhanced CT showing a pseudoaneurysm (arrow) and contrast extravasation (arrowhead). (b and c) Left renal angiograms using a 5-Fr catheter (white arrowheads) showing a pseudoaneurysm (white arrows) and contrast extravasation (black arrows). The left radial artery (black arrowheads) was punctured under ultrasound guidance (inset in B). After superselection of the bleeders with a microcatheter, embolization was performed with n-butyl cyanoacrylate (3:1, oil:NBCA) (not shown) (d) Post-embolization left renal arteriogram showing disappearance of the bleeding focus. The procedure duration was 10 minutes, and the fluoroscopic time was 3.8 minutes. The digital subtraction angiography number was 2, and the dose area product was 8,885 Gy·cm^2^. (e) Four-week follow-up ultrasound showing the left radial artery was patent. There were no differences in mean age, gender, height, weight, body mass index, systolic blood pressure, diabetes mellitus, coronary artery disease, smoking, chronic lung disease, chronic kidney disease, estimated glomerular filtration rate (eGFR), and updated Charlson comorbidity score between the TRA and TFA groups (Table 1). Prothrombin time (PT) and international normalized ratio (INR) were significantly higher in the TFA than in the TRA group (13.94±3.39 vs. 12.36±0.63 sec, p = 0.037 for PT; 1.21±0.27 vs. 1.03±0.05, p = 0.004 for INR).

Sixteen patients underwent RAE via TRA, whereas 27 underwent RAE via TFA, including two patients who underwent two TFA procedures each, for a total of 29 TFA procedures. The causes of iatrogenic renal hemorrhage in the 16 patients in the TRA group were partial nephrectomy in 11, biopsy in three, extracorporeal shock wave lithotripsy in one, and percutaneous nephrolithotomy in one. In comparison, the causes of iatrogenic renal hemorrhage in the 27 patients in the TFA group were partial nephrectomy in 15 (Fig 1), percutaneous nephrolithotomy in four, biopsy in three, radiofrequency ablation in two, percutaneous nephrostomy in two, and gold marker insertion in one. CT scans were performed within 1 day of RAE following 14 TRA and 26 TFA procedures, with all CT scans showing contrast extravasation, pseudoaneurysm, and/or hematoma.

Angiographic findings, embolization details, radiation exposure, clinical outcomes, and complications are summarized in Table 2. No differences in lesion laterality were observed between the 16 TRA and 29 TFA procedures.

**Table 2:** Angiography findings, embolization details, radiation exposure, and clinical outcomes.

	TRA (n = 16)	TFA (n = 29)	*P* value
Right:Left (Lesion laterality)	6:10	15:14	0.360
Angiography findings	PSA (n = 4)	PSA (n = 9)	0.116
	CE (n = 3)	CE (n = 7)	
	Suspicious bleeding (n = 3)	AVF (n = 4)	
	Cut-off (n = 2)	PSA/AVF (n = 3)	
	Irregularity (n = 2)	CE/PSA (n = 2)	
	PSA/AVF (n = 1)	CE/PSA/AVF (n = 2)	
	CE/AVF (n = 1)	Cut-off (n = 1)	
		Suspicious bleeding (n = 1)	
Superselection	16 (100%)	28 (96.6%)	1
Embolic materials	NBCA (n = 8)	NBCA (n = 11)	0.044
	GSP (n = 2)	NBCA/coils (n = 10)	
	NBCA/GSP (n = 2)	GSP (n = 2)	
	Coils (n = 1)	Coils/GSP (n = 2)	
	Coils/GSP (n = 1)	NBCA/GSP/coils (n = 1)	
	NBCA/GSP/coils (n = 1)	NBCA/GSP (n = 1)	
	PVA/GSP/coils (n = 1)	PVA/GSP (n = 1)	
		PVA/Coils/NBCA (n = 1)	
CM amount (mL)	156.25±30.52	145.93±40.1	0.738
Procedure duration (min)	31.31±17.52	33.97±15.97	0.469
Fluoroscopy time (min)	11.59±6.74	13.37±6.85	0.393
DSA number	4.13±1.71	5±1.89	0.148
DAP	51245.63±29199.3	70552.69±51337.9	0.349
DAP (median (IQR))	52376.5 (26580.5, 63619)	54027 (32500, 88432)	
Technical success	16 (100%)	28 (96.6%)	1
Clinical success	16 (100%)	28 (96.6%)	1
Cr. before RAE (mg/dL)	1.22±0.4	1.13±0.51	0.333
Cr. after RAE (mg/dL)	1.41±0.8	1.14±0.50	0.255
Complications	No	No	1

Results are reported as mean ± standard deviation or as number (%), unless otherwise indicated.

**Abbreviations**: AVF, arteriovenous fistula; CE, contrast extravasation; CM, contrast material; Cr, creatinine; DAP, dose area product; DSA, digital subtraction angiography; GSP, gelatin sponge particles; IQR, interquartile range; NBCA, n-butyl cyanoacrylate; PSA, pseudoaneurysm; PVA, polyvinyl alcohol; RAE, renal artery embolization; SD, standard deviation; TFA, transfemoral access; TRA, transradial access.

Angiographic findings did not differ significantly between these groups, with pseudoaneurysm and contrast extravasation being the most common in both groups (Fig 1). Superselection of the bleeding focus was achieved in 100% of the TRA and 96.6% of the TFA procedures (p>0.05). The embolic materials were very diverse, differing significantly in the two groups (p<0.05), although NBCA was the most common in both groups (Fig 1). The volume of contrast material was slightly higher in the TRA than in the TFA group (156.25±30.52 vs. 145.93±40.1 mL, p = 0.738), but the difference was not statistically significant. Procedure duration, fluoroscopy time, DSA number, and DAP were slightly lower in the TRA than in the TFA group, but these differences were not significant.

The technical and clinical success rates in the TRA and TFA groups were 100% and 96.6%, and 100% and 96.6%, respectively. One TFA procedure failed technically, with this procedure not achieving clinical success. Serum creatinine concentrations did not differ significantly in the two groups, either before or after RAE, and there was no significant difference in changes in creatinine levels after RAE in the two groups. During the 4-week follow-up period, no patient in either group experienced minor or major complications associated with RAE.

## Discussion

This study demonstrated that the technical and clinical outcomes following RAE via TRA were similar to those of RAE via TFA for the management of iatrogenic renal hemorrhage, with no access site complications, similar fluoroscopy times, and similar radiation exposure. Although embolization itself is not dependent on the route of access, TRA may have several advantages, including patient comfort and a low rate of access site complications, especially in patients with obesity or coagulopathy [5, 14].

The demographic characteristics, underlying diseases, and comorbidities of patients in the TRA and TFA groups did not differ significantly. However, PT and INR were significantly higher in the TFA group, although the means for both groups were within normal ranges. These differences in coagulation parameters were likely caused by the retrospective nature of this study. However, no patient in either group had access site complications, indicating that RAE via both TRA and TFA is safe.

Radial artery occlusion is the most common significant complication after TRA coronary interventions, with a composite of three studies in a total of 7930 patients, showing an average incidence of 1.3% (range 0.9–5.3%) [15–17]. In comparison, the incidence of radial artery occlusion in interventional radiology procedures may be similar or lower, occurring after 0.7% of 1512 TRA procedures [14]. Absence of radial artery occlusion in this study may have been due to the use of lower profile sheaths (all 5-Fr), use of nitroglycerin and verapamil as antispasmodic drugs, and application of patent hemostasis [18]. Although a combination of the antispasmodic agents such as heparin, nitroglycerin, and verapamil is most commonly used in this procedure [19], no consensus has been reached on the optimal combination of antispasmodic drugs agents. Because all patients in the present study had renal hemorrhage, heparin was omitted from the antispasmodic cocktail. Despite its omission, none of these patients experienced radial artery thrombosis or occlusion. Further research is needed to assess the usefulness of heparin in bleeding patients.

Angiographic findings did not differ in the TRA and TFA groups. By contrast, embolic materials differed significantly in the two groups. NBCA was used in 50% of TRA and 38% of TFA procedures. Moreover, the use of NBCA/coils was common in the TFA group, perhaps due mainly to operator preference. All TRA procedures and 28 of 29 TFA procedures achieved both technical and clinical success, indicating that hemostasis was well maintained in both groups regardless of embolic materials, and that technical specifications related to the access sites did not result in a difference in clinical success.

A comparison of TRA and TFA for abdominal and peripheral interventions showed that the procedure duration was significantly longer in the TRA than in the TFA group [6]. In that study, 77% of the procedures were hepatic artery interventions, including Y90 mapping, embolization, or transarterial chemoembolization. In the present study, however, in which patients underwent RAE, the procedure duration and fluoroscopy time were shorter, and the DSA number and DAP lower, in the TRA than in the TFA group, although none of these differences was statistically significant. Although radial artery puncture was likely less familiar than femoral access in TRA, the duration of RAE seems to depend on selective angiography, microcatheter selection, and embolization rather than the time from arterial access to renal artery selection. By contrast, less time may be required for renal artery selection in TRA; this may be explained by easy and stable catheter selection due to the downward angle of the renal artery in the aorta. The effect of renal artery angulation on procedure duration is consistent with the effect of angulation on renal artery cannulation time in snorkel/chimney EVAR [20]. In that study, renal artery angulation was measured at the horizontal plane perpendicular to the aortic wall at the midpoint of the renal ostium, resulting in cannulation that was significantly shorter at a greater downward (<−30°) angulation (10.9 vs. 17.3 minutes, p = 0.05) [20].

Pelvic and lower extremity interventions require long catheters and interventional devices [6, 14, 21], whereas a 125 cm long 4-Fr or 5-Fr catheter is sufficient for renal artery interventions, similar to hepatic artery interventions. Moreover, most embolization procedures do not require devices larger than the catheter profile, allowing the application of TRA without changing to a larger sheath. TRA is considered a very useful procedure for RAE because it uses microcatheters of various diameters and allows the use of a variety of embolic materials. Moreover, the procedure duration and radiation exposure are similar to those of TFA.

The present study had several limitations, including its retrospective design and small sample size, limiting the ability to detect small differences between the two groups. In addition, this study did not evaluate patient quality of life. Prospective randomized studies involving a larger number of patients are needed to compare these two procedures.

In conclusion, the present study demonstrates that RAE via TRA is feasible and safe for the management of iatrogenic renal hemorrhage. The procedure duration and radiation exposure in patients undergoing RAE via TRA were similar to those of patients undergoing RAE via TFA, suggesting that TRA may be an acceptable alternative to TFA.

## Supporting information

S1 Data Patient raw data was uploaded as a supplementary excel file. (XLSX)
